# The embryology of the retinal pigmented epithelium in dwarf geckos (Gekkota: Sphaerodactylinae): a unique developmental pattern

**DOI:** 10.1186/1471-213X-14-29

**Published:** 2014-06-30

**Authors:** Ricardo A Guerra-Fuentes, Juan D Daza, Aaron M Bauer

**Affiliations:** 1 Museu de Zoologia, Universidade de São Paulo, CP 42.494, 04218-970 São Paulo, Brazil; 2 Biology Department, Villanova University, 800 Lancaster Avenue, Villanova, PA 19085-1699, USA; 3 Department of Biological Sciences, Sam Houston State University, 1900 Avenue I, Huntsville, TX 77341-2116, USA

**Keywords:** Squamata, Eye development, Concaviclivate temporal fovea, Conjunctival papillae

## Abstract

**Background:**

The retinal pigmented epithelium (RPE) is a rounded shaped structure in almost all lizards. In the New World dwarf geckos, this structure shows an unusual morphology. In addition to this ocular character, we describe notable differences in the development of these geckos in comparison with available developmental staging tables for other geckos and squamate reptiles.

**Results:**

We identified two main patterns of development of the RPE for squamates. These patterns were mapped onto a metatree of concordant hypotheses of squamates based on molecular data. During post-ovopositional stages the representative species of sphaerodactyls exhibit a RPE layer that transforms gradually from an ovoid form into the generalized spherical form. Sphaerodactyls are the only group of squamates in which this pattern is known.

**Conclusions:**

This transition might be circumstantial evidence that the accessory RPE plays a role in providing additional protection for their apomorphic concaviclivate temporal fovea. We also report the presence of conjunctival papillae in a developmental stage prior to the formation of scleral ossicles. This developmental progression is similar to that of birds and turtles.

## Background

The New World dwarf geckos (Sphaerodactylinae) or ‘sphaerodactyls’ include about 164 species, accounting for more than 11% of the known diversity of the Gekkota [1-3]. Originally the term “sphaerodactyls” was used to refer exclusively to members of genus *Sphaerodactylus*[4-9], but it has more recently been used to include the genera *Gonatodes*, *Lepidoblepharis*, *Chatogekko*, *Coleodactylus*, *Pseudogonatodes*, and *Sphaerodactylus*[1,10,11]; in this paper, we follow the latter usage.

Sphaerodactyls are diurnal and represent some of the most extremely miniaturized extant amniotes (viz. *Sphaerodactylus ariasae* and *S. parthenopion*, [12,13]), equaled only by tiny leaf chameleons from Madagascar [14,15]. Some sphaerodactyls are climbers and utilize vertical surfaces [16,17], but the majority of them are surface dwellers, found in forest or littoral leaf litter [4,18,19]. They combine their small size with a secretive lifestyle, which is common among lizards [20] and is an ecological niche that is known to have been occupied by members of Squamata as early as 120 million years ago [21].

Sphaerodactyls are diurnal and develop a shallow, bowl-like or concaviclivate temporal fovea in the retinal tissue [22,23]. These geckos are also known to be affected by variations of environmental factors such as illumination [24]. For example, *Sphaerodactylus macrolepis* from forested habitats detects motion better in dim light than conspecifics from coastal areas, which detect movement better in much brighter conditions [25].

Binocularity (stereopsis) for deep perception in this group might also be facilitated because of their large eyes and generally narrow snouts [JDD, pers. obs.], analogous to other squamates with binocular vision, such as vine snakes [26]. Narrow snouts coupled with ocular adaptations to diurnal habits suggest that this group of geckos may exhibit some anatomical differences from other lizards, including nocturnal geckos.

Sixty years ago, Garth Underwood developed a classification of geckos using visual system characters [22,27-29]. Under this classification, sphaerodactyls were placed in a separate family based on several morphological characters, such as presence of a temporal fovea, a round pupil (elliptical with straight vertical margins when closed), and presence of a brille covering the eye [22,29], in combination with additional osteological [30] and integumentary [31] characters.

**Figure 1:**
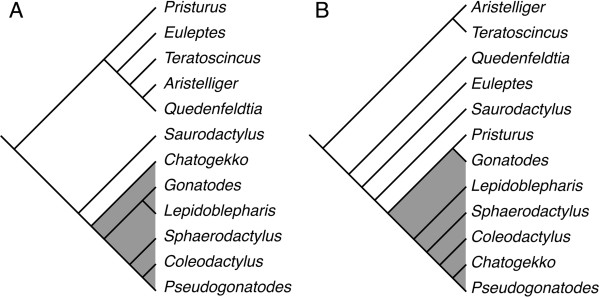
**Two alternate hypotheses for the relationships of Sphaerodactylidae. A)** Molecular hypothesis [2]; **B)** Morphological hypothesis [38]. Gray shading indicates the sphaerodactyl group.

Subsequent to Underwood’s classification of geckos [29], morphological evidence has been accumulated in support of the monophyly of the sphaerodactyls and their relationships with other gekkotan genera [11,32-39]. Recent molecular evidence has corroborated the split of sphaerodactyls from the Gekkonidae *sensu stricto* and grouped them together in an expanded Family Sphaerodactylidae with the Caribbean genus *Aristelliger* and the Old World genera *Euleptes*, *Pristurus*, *Quedenfeldtia*, *Saurodactylus* and *Teratoscincus* ([1,2], Figure 1). Although Underwood’s classification is outdated, some of the characters that he used still have taxonomic utility [40], for example, the eye morphology that he attributed to the sphaerodactyls has also been found in most of the genera recently placed in the family (e.g., *Aristelliger, Pristurus, Quedenfeldtia, Saurodactylus, Teratoscincus*).

However, the latest molecular and morphological hypotheses of relationships within the Sphaerodactylidae are not entirely congruent [2,39]. Molecular data indicate a single origin for the sphaerodactyls (Figure 1A) whereas morphology suggests that the Northeast African/Middle Eastern genus *Pristurus* is nested within the sphaerodactyls (Figure 1B). These two hypotheses also differ from the previous phylogenetic hypothesis based on morphology of the group [11].

Here we investigate in detail a preliminary observation by Werner [41] on eye development in *Sphaerodactylus argus* in postovopositional stages. Although Werner did not describe in text the changes of the eye during development, he did illustrate a remarkable change in eye morphology across the embryological stages of this species. Using Werner’s staging for *Sphaerodactylus argus*, it can be seen that in embryos at approximately stages 30–31, the outer layer of the optic cup contains an ovoid retinal pigment epithelium (henceforth RPE), extending considerably towards the posterolateral portion of the eye. After stage 31, the iris is clearly differentiated but this pigmentation extends posteriorly, forming an ovoid extension and creating the appearance of a bandit’s mask in the embryo. This posterior extension of RPE remains until approximately stage 35 when it gradually becomes less accentuated and the iris becomes more rounded (Figure five in [41]).

In this paper we present information on the embryology of sphaerodactyls, making special reference to eye development and the retinal pigmented epithelium, and contrast this with embryological information from other lizard families, including some nocturnal gekkotans. To date most contributions to the study of gekkotan ontogeny have been descriptions of the external morphology across developmental series [41-45] and osteological descriptions of a few species [46-51]. Only some studies have evaluated the phylogenetic significance of embryological data for squamate systematics [52-57]. This study explores the distribution of two contrasting patterns of development for the eye, and has the potential to provide additional morphological evidence for the understanding of the evolution of this diurnal clade of geckos.

## Methods

Obtaining complete developmental series from natural populations of geckos is difficult because of their small egg clutches. Sphaerodactylids lay a maximum of two eggs per clutch, and most sphaerodactyl species lay only one [1]. We describe morphological changes in squamate specimens during post-ovopositional stages 28–42. We mainly used the developmental diagrams of *Sphaerodactylus argus*[41] to assess the stages, which follow a previous study [58] on *Lacerta agilis.* We tried to find some agreement between these development tables and the recently described table for the eublepharid gecko *Eublepharis macularius*[45], which is based on *Gallus gallus*[59], but there are substantial differences between comparable stages among species. This observation is congruent with previous comparisons of embryos from different species in which variation in form due to allometry, heterochrony, and differences in body plan and somite number have been reported [60]. We determined the developmental stage by examining external characters such as pharyngeal clefts, eyelids, tympanic aperture, limbs, scale morphology, and pigmentation. These structures have been used in recent developmental staging studies [44,45,61-63].

Direct observations of eye development were observed in 88 embryos of nine species. We examined eye development in embryos from different sphaerodactyl species (*Chatogekko amazonicus*, *Coleodactylus meridionalis*, *Coleodactylus* sp., *Gonatodes albogularis, Gonatodes humeralis, Sphaerodactylus macrolepis*), representatives from other gekkotan clades (i.e., Gekkonidae, Phyllodactylidae), and other squamate families (Amphisbaenidae, Anguidae, Iguanidae, Polychrotidae, Scincidae, Teiidae, Tropiduridae, Dipsadidae). All squamate embryos in this study were extracted from eggs collected in the field, and cataloged at the Museu de Zoologia, Universidade de São Paulo, Brazil (MZUSP), Museo de Zoología de la Universidad de Puerto Rico, Río Piedras Campus, San Juan, Puerto Rico (UPRRP), and Aaron M. Bauer personal collection at Villanova University, USA (AMB).

The observed visual system characters and additional morphological changes were compared with previous descriptions of the embryology of other squamate groups (i.e., Agamidae, Chamaeleonidae, Dactyloidae, Eublepharidae, Scincidae, Lacertidae) based on literature sources [41,44,45,58,63-69], see Additional file 1 for details on the source of specimens. Development patterns of the REP were mapped as a single character onto a metatree of squamates using the Module Ancestral State Reconstruction (parsimony ancestral states) on Mesquite Version 2.75 [70].

Animals were euthanized according to an approved protocol for ectothermic embryo use (available at: http://www.iacuc.org). The embryos were removed from the egg and immersed in a solution of ethyl 3-aminobenzoate methanesulfonate 0.2%. This protocol of euthanasia has been approved by Comissão de Ética no Uso de Animais do Instituto Butantan (Protocol CEUAIB 944/12), Sao Pablo, Brazil and entirely meets the terms of the Institutional Animal Care and Use Committees (IACUC, U.S.A.) and the Federal Constitution (FC, Brazil) Law 11974/2008 for breeding and using animals in teaching and scientific research.

**Figure 2:**
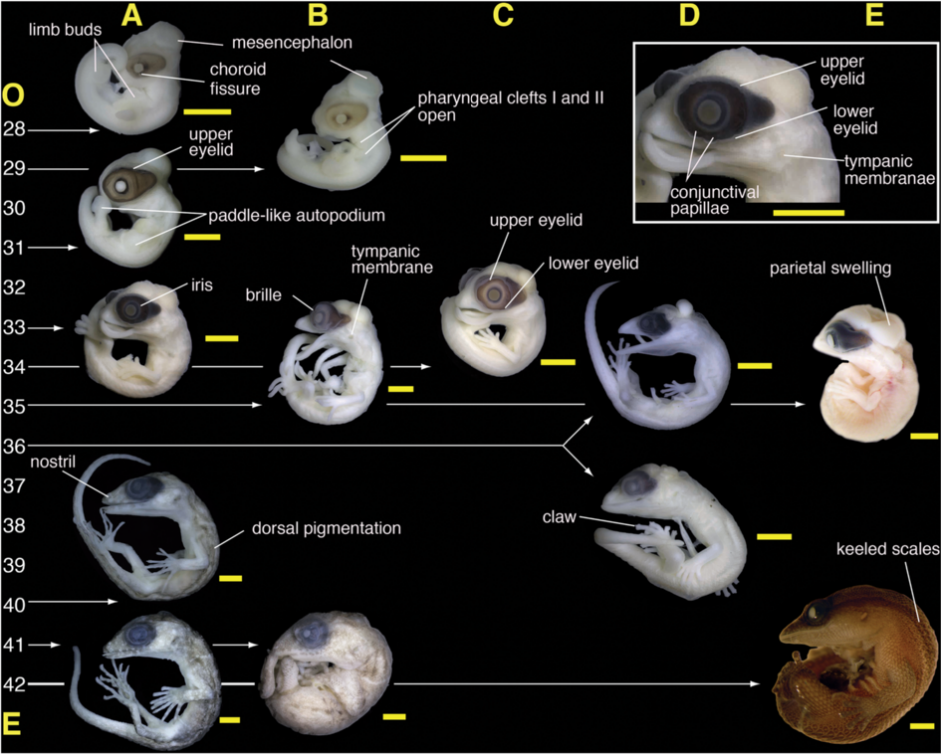
**Embryos from five species of sphaerodactyls.** Inset, eye detail at the stage 33 of *Gonatodes albogularis*. **A)***Gonatodes albogularis*; **B)***Gonatodes humeralis*; **C)***Chatogekko amazonicus;***D)***Coleodactylus meridionalis* and *Coleodactylus* sp.; **E)***Sphaerodactylus macrolepis mimetes*. Specimen numbers: A-28) MZUSP 99997; A-31) MZUSP 99998; A-33) MZUSP 99999; A-40) MZUSP 100117; A-41) MZUSP 100113; B-29) MZUSP 101107; B-35) MZUSP 101108; B-41) MZUSP 101109; C-34) MZUSP 101105; D-36, upper) MZUSP 101099; D-36, lower) MZUSP 101103; E35, E42) UPRRP uncataloged. Numbers at the left side indicate the approximated embryonic stage between oviposition **(O)** and eclosion **(E)** based on the developmental table of *Eublepharis macularius*[48]. Scale bar equals 1 mm.

Specimens were photographed at the Museu de Zoologia, Universidade de São Paulo using a modular Leica M205 stereo microscope (Leica Microsystems GmbH, Wetzlar Germany) with a digital color Leica DFC425 microscope camera. Images were processed on a desktop computer running Leica LAS Montage software. Snout-vent length (SVL) measurements were taken for each embryo. Figures in the text are numbered sequentially and include a coordinate system composed of a letter and number. Each letter represents a species, and all the embryos referable to that species are organized sequentially following the letter in their corresponding embryological stage (number in the horizontal rows), for example Figure 2A-28, refers to *Gonatodes albogularis* at stage number 28.

## Results and discussion

### Post-ovopositional embryology in sphaerodactyls with emphasis on the eye

#### Stage 28–29

Non-ocular remarks: The mesencephalon bulges at the back of the head, and the pharyngeal clefts I and II are open. At this stage, forelimb and hindlimb buds are visible, with the former larger than the latter. The paddle-shaped autopodium starts to develop by stage 29. The tip of the maxillary process reaches the level of the choroid fissure. These stages are represented by two embryos, one of *Gonatodes albogularis* (Figure 2A-28), and one of *G. humeralis* (Figure 2B-29).

Eye: An ovoid portion of the eye of specimen in these stages is covered by the RPE, including the posterior region; the choroid is pigmented by a homogeneous layer and the iris is undefined. There is a concentration of melanosomes at the posterior side of the area that corresponds to the iris. The margins of the choroid fissure are still in contact with one another, and persist until about stage 29, as seen in *G. humeralis* (Figure 2B-29).

**Figure 3:**
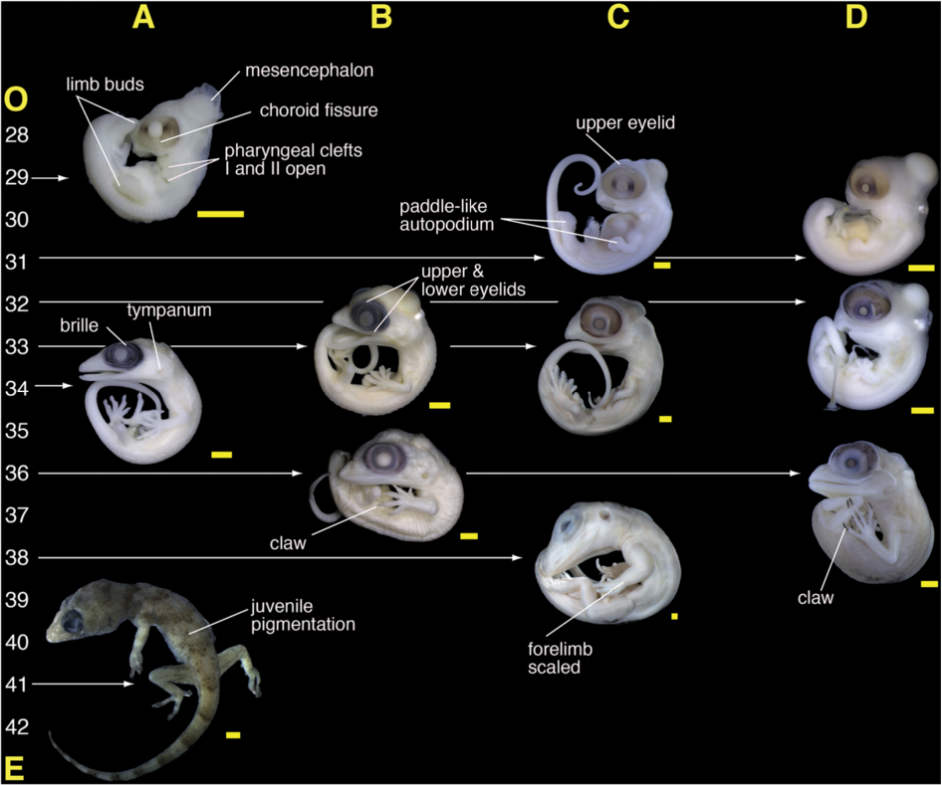
**Embryos from four species used as reference material. A)***Hemidactylus mabouia*; **B)***Phyllopezus pollicaris*; **C)***Tupinambis merianae*; **D)***Polychrus acutirostris*. Specimen numbers: A-29) MZUSP 101110; A-34) MZUSP 101102; A-41) MZUSP 101106; B-33) MZUSP 101098; B-36) MZUSP 101100; C-29) MZUSP 101126; C-33) MZUSP 101130; C-38) MZUSP 101128; D-29) MZUSP 101124; D-32) MZUSP 101122; D-36) MZUSP 101121. Numbers at the left side indicate the approximated embryonic stage between oviposition **(O)** and eclosion **(E)** based on the developmental table of *Eublepharis macularius*[45]. Scale bar equals 1 mm.

Comparison with other squamates: At this stage sphaerodactyls appear similar to *Hemidactylus mabouia* (Figure 3A-29); the choroid fissure persists, melanosomes of the RPE are concentrated on the equatorial region, and the limb buds show further development, with a paddle-shaped autopodium. In other lizards at a corresponding stage (e.g., *Eublepharis macularius*, *Lacerta agilis, Anolis sagrei*), the choroid fissure is present, and the limb buds appear as mere protuberances [45,58,64]. Sphaerodactyls differ from other lizards in the ovoid shape of the RPE of the eye (Figure 2A-28, 2B-29).

#### Stage 31–32

Non-ocular remarks: The mesencephalic bulge is less prominent, and the pharyngeal clefts I and II are closed. The maxillary process reaches the nasal capsule and the mandibular process is very close to anterior margin of the eye. Forelimbs and hindlimbs are similar to the previous stage, the autopodium is flat and paddle-like. The stylopodium and zeugopodium are undifferentiated. This stage is represented by *Gonatodes albogularis* (Figure 2A-31).Eye: The pigmentation is more uniform than previous stage, the upper eyelid is visible, and the choroid fissure is closed (Figure 2A-31). The iris starts to become delimited, the RPE larger around the iris area than in the posterior part.

Comparison with other squamates: At this stage, the RPE of sphaerodactyls has a distinct ovoid shape; *Eublepharis macularius* has been described as having a kidney-shaped eye during this stage [45], but its overall shape tends to be more rounded, as it is in other lizards (e.g., *Lacerta agilis, Anolis sagrei*) [58,63]. This can also be seen in slightly later stages of *Tupinambis merianae* and *Polychrus acutirostris* (Figure 3C-31, 3D-31). Similar to sphaerodactyls, the upper eyelid covers the dorsal surface of the eye in this stage in *Lacerta agilis*, and although the upper eyelid has been mentioned in *Eublepharis macularius* by stage 34, some thickening of the epithelium surrounding the eye in *Eublepharis macularius* can be observed as early as stage 32 (See Figure three in [45]). At stage 32 the conjunctival papillae are visible in *Polychrus acutirostris*.

#### Stage 33–34

Non-ocular remarks: The first signs of a tympanic membrane appear between these stages. The parietal area starts to become swollen. The mandibular process is projected beyond the snout tip yielding a prognathous appearance. The hemipenial buds are visible projecting from the cloaca. Digits well differentiated on both fore limbs and hind limbs, but still webbed. These stages are represented by two embryos, one of *Gonatodes albogularis* (Figure 2A-33), and one of *Chatogekko amazonicus* (Figure 2C-34).

Eye: The RPE is more uniform than in the previous stage and both the upper and lower eyelids are visible. The iris is well defined, and the eye still appears ovoid due to the posterior extension of the RPE. Fourteen conjunctival papillae are evident in *Gonatodes albogularis* at stage 33 (Figure 2A-33). One specimen (MZUSP 100000) at stage 34 had three papillae missing from the posterodorsal region of the eye. The papillae are not visible in stage 34 of *Chatogekko amazonicus* (Figure 2C-34).

Comparison with other squamates: The tympanic membrane becomes more evident by these stages, as in *Hemidactylus mabouia* and *Tupibambis merianae* (Figure 3A-33, 3C-33). In *Eublepharis macularius* the auditory meatus is apparent by stage 33, whereas this structure is illustrated earlier in *Lacerta agilis* (Stage 27 in [58]), when the limbs are merely buds. During these stages, digits of sphaerodactyls are more well-differentiated than those of *Eublepharis macularius*[45]*,* and the degree of interdigital webbing is variable. In sphaerodactyls, *Hemidactylus mabouia* and *Phyllopezus pollicaris* the digits are almost free of webbing (Figure 2A-33, 2C-34 and 3A-34, 3B-33), whereas *Tupinambis merianae* and *Polychrus acutirostris* are more extensively webbed (Figure 3C-33). Upper and lower eyelids are well developed in sphaerodactyls, *Eublepharis macularius*, *Phyllopezus pollicaris* (Figure 3B-33). The conjunctival papillae are visible in *Tupinambis merianae* (Figure 3C-33), but not in *Phyllopezus pollicaris* (Figure 3B-33).The RPE of the eye has a roughly circular shape restricted to the iris in all other squamates reviewed, whereas in sphaerodactyls the RPE remains ovoid (Figure 2A-33, 2C-34).

#### Stage 35

Non-ocular remarks: The parietal swelling is more prominent and the auditory meatus contains a better-defined tympanum than in previous stages. The mandibular process still extends beyond the snout tip. The digits are nearly free of interdigital webs, and scales start covering the epidermis of the embryos. This stage is represented by three embryos, one of *Gonatodes albogularis* (MZUSP 100001), one of *G. humeralis* (Figure 2B-35), and one of *Sphaerodactylus macrolepis* (Figure 2E-35).

Eye: In the embryo of *Gonatodes albogularis* the eyelid margin approaches to the margin of the pupil, but in *G. humeralis* and *Sphaerodactylus macrolepis* the fusion of upper and lower eyelids into an incipient brille occurs, although a faint suture remains. This character is neomorphic with respect to *E. macularius* (see Figure four in [45]) and it is produced via peramorphism with respect to all other forms with discrete eyelids. Fusion of eyelids in the Gekkota can be interpreted to have either occurred once with one reversal in the Eublepharidae (geckos with unfused eyelids) or to have been a product of two independent fusions in the Pygopodoidea and the Gekkonoidea exclusive of Eublepharidae. Fusion of eyelids has also occurred in the Xantusiidae, snakes, Amphisbaenia, and some gymnophthalmids, scincids and lacertids [52,71-73]. During this stage, the posterior part of the RPE is comparatively smaller and is concentrated around the iris; therefore the pigmented portion of the choroid changes from ovoid to more bilobular, with a smaller posterior hemisphere. In *Gonatodes albogularis* the reabsorption of the conjunctival papillae is complete.

Comparison with other squamates: By this stage sphaerodactyls and *Hemidactylus mabouia* have a well developed brille covering the eye, whereas in some geckos (e.g., eublepharids) and other squamates the eyelids remain separated and become thickened, covering the eye laterally and leaving exposed only the area near the iris. It is difficult to establish correspondence of these stages in squamate groups with attenuated bodies (e.g., amphisbaenians, snakes, and some limb-reduced or limbless squamates such as some gymnophthalmids), especially considering that many of the characters used are based on limb development. In these lineages, where the eye is also covered by a brille, the formation of this structure occurs during intermediate embryonic stages, and the complete fusion of eyelids is accomplished within a few stages [68,73,74].

Sphaerodactyls show a notable proportional reduction of the RPE posterior to the iris, but their eyes still differ considerably from other lizards with a more circular RPE.

#### Stage 36–38

Non-ocular remarks: The parietal swelling reduces, the preorbital region of the head is as long or longer than the maximum diameter of the eye, slight mandibular prognathism, digits free of webbing, phalangeal segments begin to differentiate, and initial appearance of claws. Scales cover the whole body except in the parietal region. This stage is represented by two embryos, one each of *Coleodactylus meridionalis* (Figure 2D-36 upper) and *Coleodactylus* sp. (Figure 2D-36 lower).

Eye: Fusion of eyelids is completed, forming a brille. The anterodorsal region becomes thickened, forming the supracilliary fold. The RPE posterior to the iris is further reduced in relation to the iris. The eye appears more rounded, but there is some persistent coloration posterior to the iris.

Comparison with other squamates: Integumentary characters such as the development of scales and claws accelerates in all lizards reviewed. In *Phyllopezus pollicaris* the scales cover the entire body including the parietal area (Figure 3B-36). The size difference between the preorbital region of the head and maximum diameter of the eye is not very marked in sphaerodactyls, which have a high degree of overlap among the snout bones. This overlap reduces the muzzle unit — a common process in miniaturized forms [2,75]. At approximately this stage in *Hemidactylus mabouia, Phyllopezus pollicaris, Eublepharis macularius,* and other larger lizards in which the snout is not reduced (e.g., *Tupinambis merianae*, *Polychrus acutirostris*, Figure 3C-38, 3D-36), the preorbital area starts to become proportionally larger, appearing more like it does in adults. The RPE of sphaerodactyls is more rounded and the differences with other lizards are less marked, although the RPE in other lizards is more concentrated on the iris area. Gekkotans with eyes covered by a brille differ in size from lizards with eyelids (including Eublepharidae) because the eye in the latter starts to be concealed by the thickened eyelids.

#### Stage 40

Non-ocular remarks: The external naris appears as a shallow depression, the body is completely covered by scales, and a faint juvenile pigmentation pattern is visible on the dorsum, but not extending onto the limbs. This stage is represented by *Gonatodes albogularis* (Figure 2A-40).

Eye: The eye approximates the round postnatal form, but some extension of the RPE posterior to the iris is visible.Comparison with other squamates: Lizard embryos at this point exhibit similar dull coloration. Scales around the eye are almost undifferentiated in both lizards with eyelids and ones with a brille (Figure 2A-40).

#### Stage 41

Non-ocular remarks: The external naris more distinct but remains sealed, pigmentation accentuates and extends to the limbs. This stage is represented by *Gonatodes albogularis* and *G. humeralis* (Figure 2A-41, 2B-41).

Eye: The RPE is almost round and restricted to the iris, although there is some posterior extension of the pigmented area covered by the postorbital integument. There are well-defined circumorbital scales around the brille.Comparison with other squamates: Body coloration becomes darker in all embryos. Circumorbital scales more differentiated in both lizards with eyelids and those with brilles (Figure 2A-41, 2B-41, 3A-41).

#### Stage 42

Non-ocular remarks: The external naris opens, pigmentation becomes accentuated, even extended into the abdominal areas. Egg teeth are present on the upper jaw. This stage is represented by *Sphaerodactylus macrolepis* (Figure 2E-42).

Eye: The RPE is rounded, with the normal circular pupil of sphaerodactylid geckos. At this stage there is no trace of the supracilliary spine, which is present in several sphaerodactylid genera (e.g. *Aristelliger*, *Gonatodes*, *Lepidoblepharis*, and *Sphaerodactylus*), which, based on this observation, would appear to develop after eclosion.

Comparison with other squamates: The fine details of the integument such as keeling appear during this stage. This can be seen in *Eublepharis macularius* and *Sphaerodactylus macrolepis* (Figure 2E-42). At this stage, *Sphaerodactylus macrolepis* develops keeled scales in the dorsal part of the body and limbs, but they are not elongated and imbricate as in the adults of this species [76]. The condition of unkeeled granular scales is widespread in sphaerodactyls [31,32,39], thus the presence of elongated, keeled, imbricate scales is peramorphic, and it is present in several species of *Sphaerodactylus* and in the *Chatogekko amazonicus* complex [2,77,78].

### Scleral ossicle development

Adult lizards, like many vertebrates, have a series of scleral ossicles that form a bony ring external to the optic cup [79,80] which maintains the shape of the eyeball [81,82]. In *Gonatodes albogularis, Polychrus* and *Tupinambis,* we observed discrete structures in the conjunctival region of the eyes arranged in a circle surrounding the pupillary region (thereofore identified as conjunctival papillae) at stages 32–33. In *Gonatodes albogularis* the conjunctival papillae show notable reduction between stages 33 and 34, disappearing altogether by stage 35, indicating that reabsorption took place between these stages (Table 1)*.*

**Table 1:** Number of scleral ossicles in geckos; presence and conjunctival papillae counts in each embryonic stage

**Species**	**Adult scleral ossicles**	**Developmental stage**	**Conjunctival papillae condition**
*Gonatodes albogularis*	14	Stage 31	Absent
	14	Stage 33	Present; 14 papillae
	14	Stage 34	Present; 11 papillae
	14	Stage 35	Absent
*Gonatodes humeralis*	13-14	Stage 35	Absent
*Chatogekko amazonicus*	12-14	Stage 34	Absent
*Sphaerodactylus macrolepis mimetes*	13-14	Stage 34	Absent
*Hemidactylus mabouia*	?	Stage 34	Absent
*Phyllopezus pollicaris*	14-15	Stage 33	Absent
*Polychrus acutirostris*	14	Stage 29	Absent
	14	Stage 33	Present; 11 papillae
*Tupinambis merianae*	14	Stage 29	Absent
	14	Stage 33	Present; 14 papillae

In bird and turtle embryos, the conjunctival papillae appear before the formation of scleral ossicles [83]. The intramembranous ossification of the scleral ossicles has been studied in chicken embryos [84,85], and they are found to be induced by the conjunctival papillae and, thickenings of the conjunctival epithelium [86,87]. In a recent study, the presence of the conjunctival papillae was confirmed in the turtle *Chelydra serpentina*, and it was proposed that this structure plays a similar role in the ossification of the sclerotic ring [85]. The similar position of the scleral ossicles in several groups of Tetrapoda (e.g., chickens and turtles) in conjunction with their similar developmental pattern, has led some authors to claim that they are homologous structures [86]. Our observations of the conjunctival papillae, and their transient apperance in squamate embryos adds evidence to this interpretation of the origin of scleral ossicles in reptiles.

### Implications of RPE development in dwarf geckos

**Figure 4:**
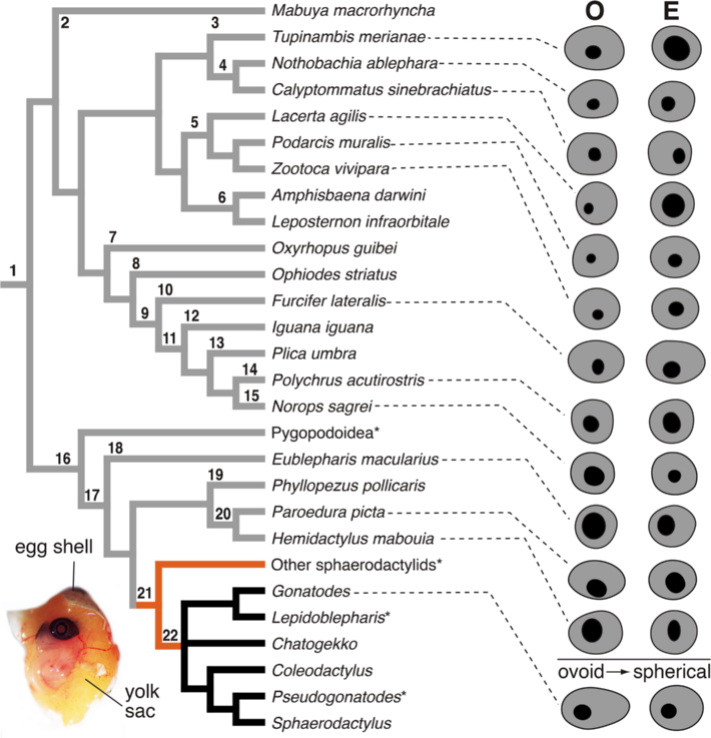
**Eye development patterns of the RPE.** The terminals in the tree include only those species for which embryological data is available. The two alternative patterns of development of RPE were mapped onto a metatree of Squamata based on molecular hypotheses from four independent studies: Lacertidae [88]; Gekkota [89]; Squamata [90,91]. Gray branches indicate species with the spherical RPE pattern, black branches ovoid → spherical RPE pattern. Asterisk indicates taxa not observed; orange branches indicate uncertain character state. Diagrams of the RPE at oviposition **(O)** and eclosion **(E)** were redrawn from published figures for those species (see text for references). Numbers on the cladogram correspond to the following taxonomic groups: 1-Squamata, 2-Scincidae, 3-Teiidae, 4-Gymnophthalmidae, 5-Lacertidae, 6-Amphisbaenidae, 7-Serpentes, 8-Anguidae, 9-Iguania, 10-Chamaeleonidae, 11-Iguanoidea, 12-Iguanidae, 13-Tropiduridae, 14-Polychrotidae, 15-Dactyloidae, 16-Gekkota, 17-Gekkonoidea, 18-Eublepharidae, 19-Phyllodactylidae, 20-Gekkonidae, 21-Sphaerodactylidae, 22-Sphaerodactylinae (sphaerodactyls). Inset, *Sphaerodactylus macrolepis mimetes* egg open to expose embryo (Stage 35).

Current observations cover most of the squamate clades, including data for at least 24 taxa represented by field-collected embryos and 11 published developmental tables. The pattern of eye development exhibited by the majority of squamates observed involves an outer layer of the optic cup with a RPE that is generally spherical throughout all embryonic stages (Figure 4). This pattern of development is regular across vertebrates [79,84]. There is, however, some variation within this widespread pattern, e.g., lacertids seem to exhibit a proportionally small pupil at oviposition (Figure 4). Likewise, the conspicuously deviant pattern observed here only among sphaerodactyl geckos is consistent among all constituent taxa sampled (Figure 4).

Sphaerodactyls develop an ovoid RPE at oviposition, which gradually changes into a spherical shape when specimens approach eclosion.

Gekkotans including sphaerodactylids have orbits that lack the postorbital bar [50,92,93]. A posteriorly open orbit, allows for an increase of eye size, although the orbit in some gekkotans is completed posteriorly by a postorbital tendon between the jugal and the postorbitofrontal, and bounded by adductor muscles [92-96]. Although a large eye has the potential to accommodate the posterior extension of RPE, eye size and the posterior extension of the RPE are independent, because the observed eye pattern was not present in other gekkotans with similarly modified skulls [41,44,45].

*Gonatodes* and *Spherodactylus* have a concaviclivate temporal fovea [22,23], a type of foveal surface that is also found in primates and some birds with a binocular field of vision. This is in contrast to other reptiles and fish that have deep foveae [97]. A concaviclivate fovea contains an additional concentration of cone cells that has also been regarded as an adaptation to diurnal habits [79,80]. Since light absorption and filtering are among the functions of the RPE [98,99], this unique development in sphaerodactyls could potentially serve as protection against light damage in these diurnal geckos. Additionally, a posterior extension of the RPE can provide extra retinal protection to the embryo prior to oviposition, which may prevent damage to the embryo from light exposure to through the thin body wall of gravid females (e.g., from basking or foraging) The latter reduction of RPE observed between stages 34 and 37 (Figure 2) can be linked to changes in eye shape, development of the brille, and origin of the integumentary pigments, which provide accessory protection to the light sensitive photoreceptor cells.

## Conclusions

The embryonic development of the eye in the Squamata offers a rich source of variation that potentially can provide new phylogenetic information. A developmental pattern in which the RPE in the outer layer of the optic cup is transformed from ovoid to circular is only known in dwarf geckos. Additional observations on genera closely related to this clade (i.e., *Aristelliger*, *Euleptes*, *Pristurus*, *Quedenfeldtia*, *Saurodactylus*, and *Teratoscincus*) are required to determine precise origin and extension of this character within the Family Sphaerodactylidae.

The development of a posterior extension of RPE is postulated as having the potential to provide an auxiliary protective function to the concaviclivate temporal foveae of dwarf geckos, especially prior to oviposition. The evidence supporting this statement is circumstantial and needs to be tested with experimental data and verified in other diurnal geckos with similar retinal structure.

## Competing interests

The authors declare that they have no competing interests.

## Authors’ contributions

RAGF, JDD and AMB made substantial intellectual contributions to this study. All authors read and approved the final manuscript.

## Supplementary Material

Additional file 1 Material examined Description of data: Material used for description and comparative purposes, includes MZUSP accession numbers and Snout Vent Length (SVL) values for each specimen. Click here for file
