# Supplementary material for: The embryology of the retinal pigmented epithelium in dwarf geckos (Gekkota: Sphaerodactylinae): a unique developmental pattern
Source: BMC Dev Biol. 2014 Jun 30;14:29. doi: 10.1186/1471-213X-14-29 (PMC4227003; doi:10.1186/1471-213X-14-29)
Supplement: Additional file 1 — Material examined Description of data: Material used for description and comparative purposes, includes MZUSP accession numbers and Snout Vent Length (SVL) values for each specimen. [file 1471-213X-14-29-S1.docx]

# Material exammined

**IGUANIDAE:** *Iguana iguana*: MZUSP 101651 (SVL = 46 mm), MZUSP 101652 (SVL = 50 mm), MZUSP 101653 (SVL = 45 mm), MZUSP 101654 (SVL = 64 mm), MZUSP 101655 (SVL = 54 mm), MZUSP 101656 (SVL = 60 mm), MZUSP 101657 (SVL = 58 mm), MZUSP 101658 (SVL = 67 mm).

POLYCHROTIDAE: *Polychrus acutirostris*: MZUSP 101121 (SVL = 28 mm), MZUSP 101122 (SVL = 21 mm), MZUSP 101123 (SVL = 33 mm), MZUSP 101124 (SVL = 19 mm), MZUSP 101125 (SVL = 37 mm).

**TROPIDURIDAE:** *Plica umbra*: MZUSP 101659 (SVL = 21 mm), MZUSP 101660 (SVL = 28 mm), MZUSP 101661 (SVL = 28 mm), MZUSP 101662 (SVL = 28 mm), MZUSP 101663 (SVL = 35 mm).

**PHYLLODACTYLIDAE:** *Phyllopezus pollicaris*: MZUSP 101098 (SVL = 21 mm), MZUSP 101100 (SVL = 28 mm), MZUSP 101475 (SVL = 28 mm), MZUSP 101476 (SVL = 31 mm), MZUSP 101477 (SVL = 30 mm), MZUSP 101478 (SVL = 30 mm).

**SPHAERODACTYLIDAE:** *Coleodactylus* sp.: MZUSP 101103 (SVL = 14 mm), *Chatogekko amazonicus*: MZUSP 101105 (SVL = 14 mm); *Coleodactylus meridionalis*: MZUSP 101099 (SVL = 13 mm)]; *Gonatodes albogularis*: MZUSP 99997 (SVL = 8 mm), MZUSP 99998 (SVL = 12 mm), MZUSP 99999 (SVL = 13 mm), MZUSP 100000 (SVL = 13 mm), MZUSP 100001(SVL = 15 mm), MZUSP 100002 (SVL = 14 mm), MZUSP 101104 (SVL = 23 mm), MZUSP 101111 (SVL = 12 mm), MZUSP 101112 (SVL = 16 mm), MZUSP 101113 (SVL = 18 mm), MZUSP 101114 (SVL = 18 mm), MZUSP 101115 (SVL = 14 mm), MZUSP 101116 (SVL = 17 mm), MZUSP 101117 (SVL = 19 mm), MZUSP 101118 (SVL = 16 mm), MZUSP 101119 (SVL = 15 mm), MZUSP 101120 (SVL = 13 mm); *Gonatodes humeralis*: MZUSP 101107 (SVL = 8 mm), MZUSP 101108 (SVL = 14 mm), MZUSP 101109 (SVL = 21 mm). *Sphaerodactylus macrolepis mimetes*: UPRRP uncataloged (SVL = 12 mm).

**GEKKONIDAE:** *Hemidactylus mabouia*: MZUSP 101101 (SVL = 23 mm), MZUSP 101102 (SVL = 22 mm), MZUSP 101106 (SVL = 25 mm), MZUSP 101110 (SVL = 13 mm).

**SCINCIDAE:** *Mabuya macrorhyncha*: MZUSP 111148 (SVL = 21 mm, TL = 18 mm), MZUSP 111176 (SVL = 20 mm), MZUSP 406095 (SVL = 14 mm), MZUSP 40790 (SVL = 25 mm), MZUSP 40801 (SVL = 28 mm), MZUSP 40803 (SVL = 34 mm).

**TEIIDAE:** *Tupinambis merianae*: MZUSP 101126 (SVL = 26 mm), MZUSP 101127 (SVL = 49 mm), MZUSP 101128 (SVL = 63 mm), MZUSP 101129 (SVL = 70 mm), MZUSP 101130 (SVL = 33 mm).

**AMPHISBAENIDAE:** *Leposternon infraorbitale*: MZUSP 99996 (SVL = 63 mm), MZUSP 100346 (SVL = 98 mm), MZUSP 100349 (SVL = 92 mm), MZUSP 100357 (SVL = 61 mm), MZUSP 100363 (SVL = 43 mm).

**ANGUIDAE:** *Ophiodes striatus*: MZUSP 101641 (SVL = 32 mm), MZUSP 101642 (SVL = 38 mm), MZUSP 101643 (SVL = 35 mm), MZUSP 101644 (SVL = 38 mm), MZUSP 101645 (SVL = 56 mm), MZUSP 101646 (SVL = 29 mm), MZUSP 101647 (SVL = 50 mm), MZUSP 101648 (SVL = 46 mm), MZUSP 101649 (SVL = 38 mm), MZUSP 101650 (SVL = 43 mm).

**DIPSADIDAE:** *Oxyrhopus guibei*: MZUSP S18720 (SVL = 59 mm), MZUSP S18721 (SVL = 107 mm), MZUSP S18722 (SVL = 156 mm), MZUSP S18723 (SVL = 150 mm), MZUSP S18724 (SVL = 83 mm), MZUSP S18770 (SVL = 192); MZUSP S18771 (SVL = 190); MZUSP S18772 (SVL = 163).
